# Inflammatory bowel disease and risk of perineal injury in primiparous vaginal births: a retrospective cohort study

**DOI:** 10.1093/ibd/izag067

**Published:** 2026-04-25

**Authors:** Itamar Gilboa, Daniel Gabbai, Noy Segal, Ronen Gold, Asnat Groutz, Avital Skornick Rapaport, Yariv Yogev, Yoav Baruch

**Affiliations:** Lis Hospital for Women’s Health, Tel Aviv Sourasky Medical Center, Tel Aviv, Israel; Gray Faculty of Medicine, Tel Aviv University, Tel Aviv, Israel; Lis Hospital for Women’s Health, Tel Aviv Sourasky Medical Center, Tel Aviv, Israel; Gray Faculty of Medicine, Tel Aviv University, Tel Aviv, Israel; Gray Faculty of Medicine, Tel Aviv University, Tel Aviv, Israel; Lis Hospital for Women’s Health, Tel Aviv Sourasky Medical Center, Tel Aviv, Israel; Gray Faculty of Medicine, Tel Aviv University, Tel Aviv, Israel; Lis Hospital for Women’s Health, Tel Aviv Sourasky Medical Center, Tel Aviv, Israel; Gray Faculty of Medicine, Tel Aviv University, Tel Aviv, Israel; Lis Hospital for Women’s Health, Tel Aviv Sourasky Medical Center, Tel Aviv, Israel; Gray Faculty of Medicine, Tel Aviv University, Tel Aviv, Israel; Lis Hospital for Women’s Health, Tel Aviv Sourasky Medical Center, Tel Aviv, Israel; Gray Faculty of Medicine, Tel Aviv University, Tel Aviv, Israel; Lis Hospital for Women’s Health, Tel Aviv Sourasky Medical Center, Tel Aviv, Israel; Gray Faculty of Medicine, Tel Aviv University, Tel Aviv, Israel

**Keywords:** inflammatory bowel disease, ulcerative colitis, Crohn’s disease, perineal trauma, obstetric anal sphincter injury

## Abstract

**Background:**

The association between inflammatory bowel disease (IBD) without active perineal disease and the risk of perineal trauma during vaginal birth remains unclear. Thus, we aimed to determine whether IBD without active perianal disease is independently associated with perineal trauma.

**Methods:**

A retrospective cohort study including all primiparous women with singleton, cephalic, term pregnancies who delivered vaginally at a tertiary medical center between 2012 and 2023. Women with IBD without active perianal disease were compared with women without IBD. Women with active perianal disease were excluded according to institutional guidelines. The primary outcomes included overall perineal injury, defined as any spontaneous perineal tear, labial laceration, obstetric anal sphincter injury (OASI), and episiotomy. Multivariable logistic regression was performed to assess the association between inflammatory bowel disease and perineal trauma, adjusting for potential confounders.

**Results:**

Among 45,250 primiparous women, 244 women (0.5%) had IBD. Overall perineal injury rates were similar between the IBD and control groups (84.0% vs. 85.4%; *P* = .553). OASI was comparable (0.4% vs. 0.9%; *P* = .728), and episiotomy rates did not differ significantly. In multivariable analysis, IBD was not associated with perineal trauma (aOR 0.82; 95% CI 0.56-1.19, *P* = .288). Independent predictors included vacuum-assisted delivery, higher birthweight, occiput posterior presentation, prolonged second stage, and epidural analgesia. Subgroup analysis showed no differences between ulcerative colitis and Crohn’s disease.

**Conclusion:**

IBD without active perianal disease does not increase the risk of perineal injury in primiparous women undergoing term vaginal delivery. Vaginal birth is an appropriate mode of delivery in this population.

Key messages **What is already known?** 

Women with inflammatory bowel disease often face uncertainty regarding the safety of vaginal delivery and the risk of perineal injury.

**What is new here?** 

In this large cohort of primiparous deliveries, IBD was not independently associated with perineal injury, including episiotomy and obstetric anal sphincter injury.

**How can this study help patient care?** 

These data support evidence-based counseling favoring planned vaginal delivery for women with IBD without active perianal disease, potentially reducing unnecessary cesarean deliveries.

## Introduction

Perineal injury is common in vaginal birth and may contribute to postpartum pain and pelvic floor morbidity.^1–3^ Inflammatory bowel disease (IBD), including ulcerative colitis and Crohn’s disease, is increasingly diagnosed in women of reproductive age.^4^^,^^5^ Concerns regarding vaginal delivery in this population stem primarily from perianal Crohn’s disease, where fistulas, abscesses, and sphincter involvement may increase the risk for obstetric injury.^6^^,^^7^ In contrast, luminal IBD does not involve perineal tissues, and current guidelines support vaginal delivery for these patients, reserving cesarean delivery for those with active perianal disease.^6^^,^^8^

Evidence on perineal outcomes in women with luminal IBD is limited and inconsistent. Some studies reported higher rates of postpartum bowel dysfunction,^9^ which may raise concern for occult obstetric anal sphincter injury or pelvic floor trauma. However, these symptoms may also reflect IBD activity and baseline bowel habits rather than delivery-related injury. In contrast, other studies found no increased risk of perineal lacerations but were limited by small sample sizes, mixed parity populations, and inadequate adjustment for key obstetric determinants such as operative vaginal delivery, fetal position, and birthweight.^10^ Moreover, most existing evidence focuses on Crohn’s disease with perianal involvement, whereas data on women with luminal disease remain limited despite representing the majority of pregnant patients with IBD. Prior data suggest that perianal phenotype may be an important determinant of perineal injury,^7^ highlighting the need for clinically well characterized studies.

Therefore, we aimed to determine whether luminal IBD without active perianal disease is independently associated with perineal trauma and obstetric anal sphincter injury among primiparous women undergoing term vaginal delivery.

## Methods

We conducted a retrospective cohort study at a university-affiliated tertiary medical center. The study population included all primiparous women with singleton, vertex-presenting pregnancies who delivered between January 2012 and December 2023. Eligible participants were primiparous women aged 18-45 years with singleton, cephalic-presenting gestations who delivered vaginally or by vacuum-assisted delivery at ≥ 37 weeks of gestation. Women with pregnancies complicated by stillbirth, those who underwent cesarean delivery, and women with active perianal disease were excluded a priori. According to institutional and international guidelines, women with active perianal disease undergo planned cesarean delivery.^6^^,^^8^

Women with inflammatory bowel disease (IBD) were identified using International Classification of Diseases codes for ulcerative colitis and Crohn’s disease. Data on IBD-related medical therapy including 5-aminosalicylates, corticosteroids, immunomodulators, and biologic agents, were extracted from the electronic medical record. In addition, medical records were reviewed for documentation of anorectal disease involvement, including active proctitis.

The comparison cohort consisted of all eligible primiparous women with no recorded diagnosis of IBD. The Sultan classification criteria was used to classify perineal tears.^11^ Prolonged second stage of labor was defined according to the presence of epidural analgesia: ≥2 hours without epidural or ≥ 3 hours with epidural anesthesia, as all participants were nulliparous. According to our institutional protocol a medio-lateral episiotomy is performed selectively according to the clinical judgment of the obstetrician or a certified midwife.

In our institution, pregnant women with inflammatory bowel disease may receive antenatal follow-up either in community obstetric clinics or in specialized clinics within our medical center. However, detailed antenatal care data for all patients were not consistently available in the obstetric database and therefore could not be analyzed.

Intrapartum care in our delivery unit follows a standardized model in which certified midwives conduct routine vaginal deliveries, while obstetricians are involved primarily in operative vaginal deliveries or cesarean deliveries. As cesarean deliveries were excluded from the cohort and operative vaginal deliveries were accounted for as a covariate in the analysis, the majority of births in this study were attended by midwives.

### Data collection

Data were retrieved from a computerized delivery room logbook containing demographic, obstetric, and clinical variables, including maternal age, body mass index (BMI), smoking status, conception by assisted reproductive technology (ART), and gestational diabetes. Intrapartum variables included induction of labor, epidural anesthesia, meconium-stained amniotic fluid, occiput posterior position, prolonged second stage, vacuum-assisted delivery, and neonatal birthweight.

### Sample size

The sample size was calculated to detect a 5% absolute difference in the rate of perineal injury between women with and without inflammatory bowel disease (IBD), assuming a two-sided significance level of 5% and a statistical power of 80%. Based on the available literature, we assumed a ratio of women without IBD to those with IBD of approximately 150:1, corresponding to an estimated IBD prevalence of 0.7%^12^ Under these assumptions, a total sample size of 36 240 women was required, including 240 women with IBD and 36 000 women without IBD.

### Statistical analysis

The categorical variables were presented as frequency and percentage. Normality of the continuous variables was tested using the Kolmogorov–Smirnov test and given as means ± standard deviation if normally distributed or median and interquartile range if normality could not be assumed. Comparisons between the groups were performed with the Student *t*-test for normally distributed continuous variables and with the Mann-Whitney rank sum test for non-normally distributed continuous variables. The Chi-square and Fisher’s exact tests were used for comparing categorical variables. Multivariate logistic regression analysis included parameters with a *P*-value < .05 in the univariate analysis, in addition to known potential confounders including pre-pregnancy BMI and occiput posterior presentation.^13^ Analyses were performed using complete-case data, and no imputation was performed. All statistical tests were two-sided, and a *P*-value < .05 was considered statistically significant. Analyses were conducted using SPSS software (version 29.0.2; IBM Corp., Armonk, NY, USA, 2023).

### Ethical considerations

This retrospective cohort study was approved by the Institutional Review Board of Tel Aviv Sourasky Medical Center (Approval No. TLV-0452-25). Due to the retrospective design and the use of anonymized routinely collected data, the requirement for informed consent was waived.

## Results

A total of 45,250 primiparous term vaginal births met inclusion criteria, including 244 women with IBD and 45,006 controls.

Perineal outcomes are presented in Table 1. Overall perineal injury rates were similar between IBD and control groups (84.0% vs. 85.4%; *P* = .553). Rates of first to second-degree tears, and labial lacerations did not differ significantly. OASI remained rare (0.4% vs. 0.9%; *P* = .728). Episiotomy rates were 40.6% among women with IBD and 36.5% among women without IBD, with no statistically significant difference between groups (*P* = .185).

**Table 1:** Perineal injury rates in women with and without inflammatory bowel disease.

Variable	**IBD *n*** = **244**	**Control *n*** = **45 006**	*P*-Value
**Overall perineal injury^a^, *n* (%)**	205 (84%)	38 419 (85.4%)	.553
**Labial tear, *n* (%)**	18 (7.4%)	4385 (9.7%)	.214
**OASI, *n* (%)**	1 (0.4%)	380 (0.85%)	.728
**Any type of perineal tear, *n* (%)**	122 (50%)	24 362 (54.1%)	.197
** 1st degree, *n* (%)**	53 (43.4%)	10 553 (43.3%)	.680
** 2nd degree, *n* (%)**	65 (53.3%)	12 377 (50.8%)	
** 1st or 2nd degree not specified, *n* (%)**	3 (2.5%)	1052 (4.3%)	
** 3rd degree, *n* (%)**	1 (0.8%)	356 (1.5%)	
** 4th degree, *n* (%)**	0 (0.0%)	24 (0.1%)	
**Episiotomy, *n* (%)**	99 (40.6%)	16 416 (36.5%)	.185

Perineal injury patterns among women with inflammatory bowel disease (IBD) compared with controls. Variables are presented as number (percentage).

Perineal lacerations are categorized according to the Sultan classification.

Unspecified non-severe tears include first- or second-degree lacerations that were not precisely classified but did not involve the anal sphincter.

Abbreviation: *OASI = *obstetric anal sphincter injuries (third- and fourth-degree tears).

a Overall perineal injury included any spontaneous tear, labial tear, and/or episiotomy.

Maternal and labor characteristics are summarized in Table 2. Women with IBD were slightly older than controls (Mean 31.6 ± 4.2 years vs. 30.8 ± 4.4; *P* = .008). No significant differences were observed in BMI, smoking status, conception by ART, gestational diabetes, occiput posterior position, vacuum-assisted delivery, or newborn birthweight.

**Table 2:** Maternal and labor characteristics in women with and without inflammatory bowel disease.

Variable	**IBD *N*** = **244**	**Control *N*** = **45 006**	*P*-Value
**Maternal age (years), mean (±SD)**	31.6 (±4.3)	30.8(±4.4)	**.008**
**Smoking, *n* (%)**	6 (2.6%)	1540 (3.5%)	.431
**ART, *n* (%)**	32 (13.7%)	5218 (12.2%)	.461
**Gestational age, median (IQR)**	39.7 (39-40.6)	39.8 (39-40.5)	.334
**BMI (kg/m²), median (IQR)**	21.4 (19.6-23.6)	21.4 (19.7-23.6)	.921
**Gestational diabetes, *n* (%)**	23 (9.4%)	4470 (9.9%)	.792
**Induction of labor, *n* (%)**	76 (31.3%)	9368 (21.0%)	**<.001**
**Epidural anesthesia, *n* (%)**	213 (87.3%)	36 667 (81.5%)	**.019**
**Occiput posterior position, *n* (%)**	9 (3.7%)	1490 (3.3%)	.743
**Meconium stain amniotic fluid, *n* (%)**	52 (21.3%)	8921 (19.8%)	.536
**Prolonged second stage, *n* (%)**	44 (18.8%)	5923 (13.6%)	**.021**
**Vacuum-assisted delivery, *n* (%)**	54 (22.1%)	8792 (19.5%)	.308
**Birthweight (g), mean (±SD)**	3202 (±366)	3220 (±391)	.458

Maternal demographics and intrapartum characteristics of women with inflammatory bowel disease (IBD) compared with controls. Continuous variables are presented as median (interquartile range) or mean ± standard deviation, as appropriate. Categorical variables are presented as number (percentage). *P*-values represent comparisons between groups. Significant differences (*P* < .05) appear in bold.

Abbreviations: *BMI* = body mass index; *ART* = assisted reproductive technology; *OP* = occiput posterior presentation.

Induction of labor was more common among women with IBD (31.3% vs. 21.0%; *P* < .001), and prolonged second stage occurred slightly more frequently (18.8% vs. 13.6%; *P* = .021). Epidural use was also higher in the IBD group (87.3% vs. 81.5%; *P* = .019). All other variables were comparable.

Medication exposure by IBD subtype is shown in Table 3. Women with ulcerative colitis were more frequently treated with 5-ASA (57.1% vs. 19.4%; *P* < .001), whereas anti-TNF therapy was more common in Crohn’s disease (23.0% vs. 7.6%; *P* = .001). Biologic therapy (Stelara, Entyvio) was infrequent and similar between groups.

**Table 3:** Medical therapy in ulcerative colitis and Crohn’s disease.

variable	**Crohn’s disease *n*** = **139**	**Ulcerative colitis *n*** = **105**	*P*-Value
**Any pharmacological Treatment, *n* (%)**	77 (55.4%)	69 (65.7%)	.104
** Single therapy, *n* (%)**	65 (84.4%)	58 (84.1%)	.120
** Dual therapy, *n* (%)**	12 (15.6%)	11(15.9%)	
**Prednisone, *n* (%)**	6 (4.3%)	5 (4.85)	1.000
**5-ASA, *n* (%)**	27 (19.4%)	60 (57.1%)	**<.001**
**Anti TNF, *n* (%)**	32 (23.0%)	8 (7.6%)	**.001**
**Thiopurine *n* (%)**	17 (12.2%)	6 (5.7%)	.085
**Biologic (Stelara, Entyvio), *n* (%)**	7 (5.0%)	1 (1.0%)	.143

Comparison of medical therapy use between women with ulcerative colitis and Crohn’s disease. Categorical variables are presented as number (percentage). *P*-values compare UC vs. Crohn’s disease. Significant differences (*P* < .05) appear in bold.

Abbreviations: *5-ASA* **=** 5-aminosalicylic acid; *Anti-TNF* = anti–tumor necrosis factor therapy; *Biologic* = ustekinumab or vedolizumab.

Multivariable logistic regression (Table 4) demonstrated that IBD was not associated with perineal trauma (aOR 0.82, 95% CI 0.56-1.19; *P* = .288). In contrast, several obstetric factors were independently associated with increased perineal injury risk. These included occiput posterior presentation (OR 1.43, 95% CI 1.17-1.76; *P* < .001), induction of labor (OR 1.29, 95% CI 1.19-1.39; *P* < .001), epidural anesthesia (OR 1.48, 95% CI 1.38-1.59; *P* < .001), vacuum-assisted delivery (OR 3.22, 95% CI 2.90-3.58; *P* < .001), and increasing newborn birthweight (OR 2.06 per kg, 95% CI 1.91-2.22; *P* < .001). Maternal age and BMI also demonstrated small but statistically significant associations with perineal trauma.

**Table 4:** Multivariate logistic regression for risk factors for perineal injury in primiparous women.

Variable	Or	95% CI
**IBD**	0.82	0.56-1.19
**Age (years)**	0.99	0.98-1.00
**BMI**	1.03	1.02-1.03
**Induction of labor**	1.29	1.19-1.39
**Occiput posterior position**	1.43	1.17-1.76
**Prolonged second stage**	1.03	0.94-1.14
**Epidural anesthesia**	1.48	1.38-1.59
**Vacuum-assisted delivery**	3.22	2.90-3.58
**Birthweight (per additional kg)**	2.06	1.91-2.22

Multivariable logistic regression evaluating independent risk factors for perineal injury among primiparous women undergoing term vaginal delivery. Odds ratios (OR) are presented with 95% confidence intervals (CI).

Abbreviations: *IBD* = inflammatory bowel disease; *BMI* **=** body mass index.

In a subgroup analysis comparing Crohn’s disease (*n* = 139) and ulcerative colitis (*n* = 105), no significant differences were observed in maternal characteristics, intrapartum variables, perineal outcomes, or OASI rates (Table 5).

**Table 5:** Maternal, intrapartum, and perineal outcomes in women with crohn’s disease compared with ulcerative colitis.

Variable	**Crohn’s disease *n*** = **139**	**Ulcerative colitis *N*** = **105**	*P*-Value
**Age, mean (SD)**	31.1 (±4.2)	31.4 (±4.4)	.707
**Smoking, *n* (%)**	4 (3.0%)	2 (2.0%)	.701
**Gestational age at birth, median (IQR)**	39.7 (38.7-40.4)	40 (39.1-40.7)	.063
**Gestational diabetes mellitus, *n* (%)**	14 (10.1%)	9 (8.6%)	.691
**Pregnancy by ART, *n* (%)**	17 (12.9%)	15 (14.9%)	.665
**Pre-pregnancy weight (kg), median (IQR)**	58 (52-65)	56 (52-62)	.156
**BMI (kg/m²), median (IQR)**	21.9 (19.7-23.9)	21 (19.5-22.9)	.117
**Induction of labor, *n* (%)**	42 (30.4%)	34 (32.4%)	.746
**Epidural anesthesia, *n* (%)**	124 (89.2%)	89 (84.8%)	.302
**Prolonged second stage, *n* (%)**	28 (21.2%)	16 (15.7%)	.283
**Meconium stain amniotic fluid, *n* (%)**	30 (21.6%)	22 (21.0%)	.905
**Occipito-posterior presentation, *n* (%)**	8 (5.8%)	1 (1.0%)	.082
**Vacuum-assisted delivery, *n* (%)**	30 (21.6%)	24 (22.9%)	.812
**Birthweight (g), mean (SD)**	3217 (±384)	3184 (±342)	.485
**Overall perineal injury, *n* (%)**	116 (83.5%)	89 (84.8%)	.782
**Episiotomy, *n* (%)**	54 (38.8%)	45 (42.9%)	.528
**Labial tear, *n* (%)**	12 (8.6%)	6 (5.7%)	.388
**Perineal tear, *n* (%)**	71 (51.1%)	51 (48.6%)	.698
** 1st degree, *n* (%)**	30 (42.2%)	23 (45.1%)	.674
** 2nd degree, *n* (%)**	37 (52.1%)	28 (54.9%)	—
** 1st or 2nd not specified, *n* (%)**	3 (4.2%)	0 (0.00%)	—
** 3rd degree, *n* (%)**	1 (1.4%)	0 (0.00%)	—
** 4th degree, *n* (%)**	0 (0.00%)	0 (0.00%)	—
**OASI, *n* (%)**	1 (0.7%)	0 (0.0%)	>.999

Comparison of maternal characteristics, labor course, and perineal injury rates between women with ulcerative colitis and Crohn’s disease.

Continuous variables are presented as median (interquartile range) or mean ± standard deviation, as appropriate.

Categorical variables are presented as number (percentage). *P*-Values represent comparisons between UC and Crohn’s disease. Significant differences (*P* < .05) appear in bold.

Abbreviations: *BMI* **=** body mass index; *ART* **=** assisted reproductive technology; *OASI* **=** obstetric anal sphincter injury.

Among women with inflammatory bowel disease, 31 (12.7%) had a history of prior bowel surgery. Most procedures were performed for Crohn’s disease and involved ileocecal or terminal ileum resections (*n* = 21). Additional procedures included right hemicolectomy (*n* = 3), segmental small bowel resections (*n* = 4). Subtotal colectomy for UC was recorded in two patients (*n* = 2), and in one patient a total colectomy for UC had been performed, this was the only case in which an ileostomy was present at the time of delivery. Importantly, no patients had undergone ileal pouch–anal anastomosis (J-pouch). Several patients also underwent procedures related to fistulizing disease (*n* = 3). Obstetric outcomes were comparable between women with and without prior bowel surgery, with no significant differences observed in rates of perineal tears, episiotomy, or obstetric anal sphincter injury (Supplementary Table S1).

## Discussion

### Principal findings

In this large retrospective cohort of primiparous women delivering at term, our main findings were:

1. Inflammatory bowel disease (IBD) without active perianal involvement was not associated with increased perineal trauma or obstetric anal sphincter injury (OASI).
2. Perineal injury rates were nearly identical between women with and without IBD, and after adjustment for comprehensive maternal, and obstetric factors, IBD remained unrelated to perineal trauma risk.
3. Other obstetric determinants, such as occiput posterior, induction of labor, epidural analgesia, VAD, and increasing newborn birthweight rather than gastrointestinal disease were significantly associated with perineal injury.

### Results in the context of what is known

Concerns regarding childbirth and perineal injury in IBD predominantly stem from the perianal phenotype of Crohn’s disease, characterized by deep tissue inflammation, fistulas, and abscesses—conditions that understandably increase vulnerability to sphincter trauma during vaginal delivery.^4^^,^^6^^,^^14^ Accordingly, current guidelines recommend cesarean delivery for women with active perianal Crohn’s disease to avoid exacerbation of local disease and risk to sphincter integrity.^8^

The existing literature addressing perineal outcomes in IBD has been inconsistent. Studies reporting increased postpartum fecal incontinence in IBD have been confounded by disease activity rather than obstetric injury,^9^^,^^15^^,^^16^ while previous studies have suggested a higher risk of fecal incontinence in women with IBD^17^ and perineal injury.^18^

Contemporary guidelines emphasize that healed or inactive perianal disease does not independently increase the risk of severe perineal tears or incontinence after vaginal delivery, risk is driven almost entirely by the presence of active perianal involvement at the time of birth.^8^^,^^14^^,^^19^

A large population-based study by Hatch et al. of 2882 women with Crohn’s disease identified perianal disease and smoking, but not Crohn’s disease itself, as independent predictors of fourth-degree lacerations, whereas episiotomy was associated with lower risk.^7^ These data support the concept that excess risk of severe obstetric trauma in IBD is largely phenotype-driven, primarily related to active perianal involvement rather than the diagnosis of Crohn’s disease alone. Consistent with this paradigm, our findings extend these observations by focusing specifically on women with IBD without active perianal disease and demonstrating comparable rates of overall perineal trauma and OASI relative to women without IBD.

Lever et al. evaluated perineal outcomes in a single-center cohort of 179 women with IBD, of whom 30.2% delivered by cesarean delivery, and reported no difference in overall or severe perineal lacerations compared with 31 258 controls.^10^ However, their analysis included mixed parity and lacked detailed intrapartum information, limiting adjustment for key obstetric determinants of perineal injury. More broadly, administrative datasets and population-based sources often lack detailed intrapartum characterization and may be affected by coding-related misclassification. Unlike such data sources, our cohort enabled evaluation within a clinically well-defined obstetric population with detailed intrapartum variables, strengthening the inference that perineal outcomes in luminal IBD are primarily determined by intrapartum mechanical factors rather than gastrointestinal diagnosis.

With respect to episiotomy, previous data suggests that women with IBD but without perianal disease have a risk profile similar to the general obstetric population.^9^^,^^10^ Episiotomy is discouraged only in women with active or complex perianal Crohn’s disease, where minimizing perineal trauma is critical to prevent recurrent inflammation, sphincter disruption, and later incontinence.^4^

Although episiotomy rates in our cohort were relatively high, this should be understood within the context of a population composed exclusively of primiparous women, who represent a high-risk group with increased likelihood of operative delivery and perineal repair.^13^^,^^20^ When compared with the literature, our rates fall squarely within the expected range, with episiotomy reported in 40%-50% of operative vaginal births and up to 89% in certain clinical settings.^21^ Importantly, and consistent with prior data, women with IBD without perianal disease exhibited episiotomy and perineal injury rates similar to those of controls.^7^ Together, these observations indicate that our episiotomy rates reflect contemporary obstetric practice in nulliparous populations and did not differ between study groups, reinforcing that IBD alone does not influence perineal management or outcomes during childbirth.

Differences in medication exposure between ulcerative colitis and Crohn’s disease in our cohort largely reflect established therapeutic practices. In particular, the low frequency of vedolizumab and ustekinumab exposure likely reflects prescribing patterns over the study period and the limited uptake of these newer biologic agents during earlier years.

Furthermore, consistent with our results, induction of labor, vacuum-assisted delivery, epidural anesthesia, occiput posterior presentation, and increased birthweight are consistently associated with higher risks of perineal injury.^13^^,^^22–25^ The slightly higher rates of induction and epidural use observed among women with IBD in our cohort may reflect differences in intrapartum management and increased obstetric surveillance in pregnancies complicated by chronic medical conditions rather than a direct effect of IBD itself. Taken together, available evidence and our findings may indicate that the risk of perineal injury in women with IBD is determined predominantly by obstetric factors and the presence of active perianal disease rather than by the diagnosis of IBD alone.

### Clinical implications

For primiparous women with luminal ulcerative colitis or non-perianal Crohn’s disease, our findings provide reassurance that vaginal delivery does not increase the risk of perineal trauma or OASI compared with women without IBD. These results support guideline-based delivery counseling and may help reduce non–obstetric-indicated cesarean delivery driven by concern rather than evidence.

### Research implications

Future research should include consistent measures of IBD activity, since disease severity may affect postpartum bowel symptoms regardless of delivery mode. Larger, multicenter studies are needed to better evaluate if specific IBD subtypes or medical treatments influence perineal risk. Prospective designs following women through pregnancy and after birth would help determine whether IBD impacts pelvic floor recovery or bowel function over time. Overall, more detailed clinical data will allow clearer risk assessment and better delivery counseling for women with IBD.

### Strengths and limitations

This study has several important strengths. We analyzed a large cohort of primiparous women undergoing term vaginal birth, enabling estimation of perineal outcomes in women with IBD compared with the general obstetric population. Restriction to primiparous women minimized confounding by parity, a key determinant of perineal trauma, thereby strengthening internal validity. In addition, women with active perianal disease were excluded according to institutional and guideline-based practice, allowing focused evaluation of luminal IBD, which represents the majority of pregnant patients with IBD. The availability of detailed intrapartum variables, including induction of labor, epidural anesthesia, fetal occiput posterior position, prolonged second stage, operative vaginal delivery, and neonatal birthweight, enabled robust adjustment for established obstetric determinants of perineal injury. In addition, inclusion of IBD subtype and medication exposure enhanced the clinical interpretability of the findings within contemporary treatment paradigms. Finally, anorectal disease involvement, including active proctitis, was specifically assessed as part of the clinical evaluation of perineal disease in the cohort, and no cases of active proctitis were identified among the women included in this study.

Several limitations should be acknowledged. The retrospective design limits causal inference and may be subject to residual confounding and outcome misclassification. Standardized measures of overall IBD disease activity, such as validated clinical activity indices, biomarkers, or endoscopic assessments, were not consistently available in the obstetric database and therefore could not be incorporated into the analysis. Additionally, the use of 5-aminosalicylates recorded among a small number of patients with Crohn’s disease may reflect treatment initiated during earlier stages of diagnostic evaluation before disease subtype was definitively established, or medications that remained listed in the medical record despite no longer being part of the active treatment regimen. This represents a limitation inherent to retrospective data extraction. Medication-specific analyses were also constrained by heterogeneity and small numbers within individual treatment subgroups, reducing statistical power for therapy-level inferences. Although OASI rates were comparable between groups, the overall incidence of this outcome was low, limiting statistical power to detect small differences. Finally, the single-center design may limit the generalizability of our findings to other healthcare systems with different obstetric practice patterns.

### Conclusion

In this large cohort of primiparous women delivering at term, luminal IBD without active perianal disease was not associated with increased perineal trauma or OASI. Perineal injury patterns were comparable between women with and without IBD, and risk appeared to be primarily driven by intrapartum mechanical obstetric factors rather than by IBD itself. These findings reinforce current recommendations that vaginal delivery is appropriate and safe for women with IBD in the absence of active perianal involvement and provide supportive evidence for counseling and delivery planning in this population.

## Supplementary Material

izag067_Supplementary_Data

## Data Availability

The datasets generated and/or analyzed during the current study are available from the corresponding author upon reasonable request.

## References

[1] Smith LA , Price N, Simonite V, Burns EE. Incidence of and risk factors for perineal trauma: a prospective observational study. BMC Pregnancy Childbirth. 2013;13:59. 10.1186/1471-2393-13-59 23497085 PMC3599825

[2] Okeahialam NA , Taithongchai A, Thakar R, Sultan AH. Corrigendum to “The incidence of anal incontinence following obstetric anal sphincter injury graded using the Sultan classification: A network meta-analysis” [American Journal of Obstetrics and Gynecology 228/6 (2023) 675-688]. Am J Obstet Gynecol. 2024;230:581. 10.1016/j.ajog.2022.11.1279 38373865

[3] Gommesen D , Nohr EA, Drue HC, Qvist N, Rasch V. Obstetric perineal tears: risk factors, wound infection and dehiscence: a prospective cohort study. Arch Gynecol Obstet. 2019;300:67-77. 10.1007/s00404-019-05165-1 31004221

[4] Shmidt E , Dubinsky MC. Inflammatory bowel disease and pregnancy. Am J Gastroenterol. 2022;117:60-68. 10.14309/ajg.0000000000001963 36194035

[5] Dolinger M , Torres J, Vermeire S. Crohn’s disease. Lancet. 2024;403:1177-1191. 10.1016/S0140-6736(23)02586-2 38437854

[6] Kothari S , Afshar Y, Friedman LS, Ahn J. AGA clinical practice update on pregnancy-related gastrointestinal and liver disease: expert review. Gastroenterology. 2024;167:1033-1045. 10.1053/j.gastro.2024.06.014 39140906

[7] Hatch Q , Champagne BJ, Maykel JA, et al Crohn’s disease and pregnancy: the impact of perianal disease on delivery methods and complications. Dis Colon Rectum. 2014;57:174-178. 10.1097/DCR.0b013e3182a41381 24401878

[8] Mahadevan U , Seow CH, Barnes EL, et al Global consensus statement on the management of pregnancy in inflammatory bowel disease. Am J Gastroenterol. 2026;121:31-79. 10.14309/ajg.0000000000003651 40862489

[9] Brochard C , Siproudhis L, Levêque J, et al Factors associated with fecal incontinence in women of childbearing age with Crohn’s disease. Inflamm Bowel Dis. 2017;23:775-780. 10.1097/MIB.0000000000001056 28394805

[10] Lever G , Chipeta H, Glanville T, Selinger C. Perineal outcomes after delivery in 179 mothers with inflammatory bowel disease compared to 31 258 controls: a single-centre cohort study. J Crohns Colitis. 2022;16:511-514. 10.1093/ecco-jcc/jjab151 34463338

[11] Pundir J , Coomarasamy A. Management of Third- and Fourth-Degree Perineal Tears. Obstetrics: Evidence-based Algorithms. Cambridge University Press; 2016:258-2261.

[12] Barnes EL , Nowell WB, Venkatachalam S, Dobes A, Kappelman MD. Racial and ethnic distribution of inflammatory bowel disease in the united states. Inflamm Bowel Dis. 2022;28:983-987. 10.1093/ibd/izab219 34473272

[13] Okeahialam NA , Sultan AH, Thakar R. The prevention of perineal trauma during vaginal birth. Am J Obstet Gynecol. 2024;230:S991-S1004. 10.1016/j.ajog.2022.06.021 37635056

[14] Nielsen OH , Gubatan JM, Kolho K-L, Streett SE, Maxwell C. Updates on the management of inflammatory bowel disease from periconception to pregnancy and lactation. Lancet. 2024;403:1291-1303. 10.1016/S0140-6736(24)00052-7 38458222

[15] Gu P , Kuenzig ME, Kaplan GG, Pimentel M, Rezaie A. Fecal incontinence in inflammatory bowel disease: a systematic review and meta-analysis. Inflamm Bowel Dis. 2018;24:1280-1290. 10.1093/ibd/izx109 29617820

[16] Johannessen HH , Mørkved S, Stordahl A, Wibe A, Falk RS. Evolution and risk factors of anal incontinence during the first 6 years after first delivery: a prospective cohort study. BJOG. 2020;127:1499-1506. 10.1111/1471-0528.16322 32418309

[17] Ong JPL , Edwards GJ, Allison MC. Mode of delivery and risk of fecal incontinence in women with or without inflammatory bowel disease: questionnaire survey. Inflamm Bowel Dis. 2007;13:1391-1394. 10.1002/ibd.20208 17576117

[18] Brandt LJ , Estabrook SG, Reinus JF. Results of a survey to evaluate whether vaginal delivery and episiotomy lead to perineal involvement in women with Crohn’s disease. Am J Gastroenterol. 1995;90:1918-1922. 7484992

[19] Nguyen GC , Seow CH, Maxwell C, et al The toronto consensus statements for the management of inflammatory bowel disease in pregnancy. Gastroenterology. 2016;150:734-757.e1. 10.1053/j.gastro.2015.12.003 26688268

[20] Muraca GM , Liu S, Sabr Y, et al Episiotomy use among vaginal deliveries and the association with anal sphincter injury: a population-based retrospective cohort study. CMAJ. 2019;191:E1149-E1158. 10.1503/cmaj.190366 31636163 PMC6805174

[21] Knight M , Chiocchia V, Partlett C, et al Prophylactic antibiotics in the prevention of infection after operative vaginal delivery (ANODE): a multicentre randomised controlled trial. Lancet. 2019;393:2395-2403. 10.1016/S0140-6736(19)30773-1 31097213 PMC6584562

[22] Pergialiotis V , Bellos I, Antsaklis A, Papapanagiotou A, Loutradis D, Daskalakis G. Maternal and neonatal outcomes following a prolonged second stage of labor: a meta-analysis of observational studies. Eur J Obstet Gynecol Reprod Biol. 2020;252:62-69. 10.1016/j.ejogrb.2020.06.018 32570187

[23] Fitzpatrick M , McQuillan K, O’Herlihy C. Influence of persistent occiput posterior position on delivery outcome. Obstet Gynecol. 2001;98:1027-1031. 10.1097/00006250-200112000-00007 11755548

[24] Dominsky O , Attali E, Amikam U, et al The association between epidural analgesia and perineal injury in primiparous women: a propensity score-matched cohort study. Int J Gynaecol Obstet. 2026;173:144-150. 10.1002/ijgo.70578 41048114 PMC12988395

[25] Gilboa I , Gabbai D, Attali E, et al Impact of gestational weight gain on perineal injury and obstetric anal sphincter injury among underweight primiparous women. Int J Gynaecol Obstet. 10.1002/ijgo.70788 2025 PMC13278628 41476389

