# Supplementary material for: Inflammatory bowel disease and risk of perineal injury in primiparous vaginal births: a retrospective cohort study
Source: Inflamm Bowel Dis. 2026 Apr 25;32(8):1524–31. doi: 10.1093/ibd/izag067 (PMC13414543; doi:10.1093/ibd/izag067)
Supplement: izag067_Supplementary_Data [file izag067_supplementary_data.docx]

**Supplementary Table S1. History of Bowel Surgery and Perineal Outcomes in**

**Women with IBD**

| **P-value** | **No prior surgery (n=213)** | **Prior bowel surgery (n=31)** | **Variable** |
| --- | --- | --- | --- |
| 0.601 | 180 (84.6%) | 25 (80.6%) | Overall perineal injury, n (%) |
| 0.578 | 85 (39.9%) | 14 (45.2%) | Episiotomy, n (%) |
| 0.140 | 18 (8.5%) | 0 (0.0%) | Labial tear, n (%) |
| 0.735 | 110 (51.6%) | 15 (48.4%) | Perineal tear, n (%) |
| — | 49 (23.0%) | 4 (12.9%) | 1^st^ Degree, n (%) |
| — | 55 (25.8%) | 10 (32.3%) | 2^nd^ Degree, n (%) |
| — | 3 (1.4%) | 0 (0.0%) | 1^st^ or 2^nd^ Not specified, n (%) |
| — | 0 (0.0%) | 1 (3.2%) | 3^rd^ Degree, n (%) |
| — | 0 (0.0%) | 0 (0.0%) | 4^th^ Degree, n (%) |
| 0.127 | 0 (0.0%) | 1 (3.2%) | OASI, n (%) |

Comparison of perineal outcomes among women with inflammatory bowel disease (IBD)

According to history of prior bowel surgery. Variables are presented as number (percentage).

P values represent comparisons between women with and without prior bowel surgery.

Perineal lacerations were classified according to the Sultan classification.

Overall perineal injury includes any spontaneous perineal tear, labial laceration, and/or

episiotomy.

**OASI** = obstetric anal sphincter injury.
